# Cross-validation of the reduced form of the Food Craving Questionnaire-Trait using confirmatory factor analysis

**DOI:** 10.3389/fpsyg.2015.00433

**Published:** 2015-04-13

**Authors:** Luca Iani, Claudio Barbaranelli, Caterina Lombardo

**Affiliations:** ^1^Department of Human Sciences, European University of RomeRome, Italy; ^2^Department of Psychology, Sapienza – Università di RomaRome, Italy

**Keywords:** measurement, reliability, validity, food craving questionnaire, confirmatory factor analysis, cross-validation

## Abstract

**Objective**: The Food Craving Questionnaire-Trait (FCQ-T) is commonly used to assess habitual food cravings among individuals. Previous studies have shown that a brief version of this instrument (FCQ-T-r) has good reliability and validity. This article is the first to use Confirmatory factor analysis to examine the psychometric properties of the FCQ-T-r in a cross-validation study.

**Method:** Habitual food cravings, as well as emotion regulation strategies, affective states, and disordered eating behaviors, were investigated in two independent samples of non-clinical adult volunteers (Sample 1: *N* = 368; Sample 2: *N* = 246). Confirmatory factor analyses were conducted to simultaneously test model fit statistics and dimensionality of the instrument. FCQ-T-r reliability was assessed by computing the composite reliability coefficient.

**Results**: Analysis supported the unidimensional structure of the scale and fit indices were acceptable for both samples. The FCQ-T-r showed excellent reliability and moderate to high correlations with negative affect and disordered eating.

**Conclusion**: Our results indicate that the FCQ-T-r scores can be reliably used to assess habitual cravings in an Italian non-clinical sample of adults. The robustness of these results is tested by a cross-validation of the model using two independent samples. Further research is required to expand on these findings, particularly in children and adolescents.

## Introduction

Craving for food has received relevant attention among scholars in the last few years. Although there is no consensual agreement on the definition of food craving, most authors describe it as physiological and psychological motivational states that promote ingestive behaviors (Weingarten and Elston, 1990; Cepeda-Benito et al., 2000a,b; Cepeda-Benito et al., 2003). Food craving has been traditionally defined as a strong or intense desire to eat and, more recently, as an intense desire to eat a particular food that is difficult to resist (Rogers and Smit, 2000; White et al., 2002). Strength of desire and specificity toward a particular food seem to be the core components of food craving (Hill, 2007).

Food cravings often co-occur with hunger; however, an energy deficit is not necessary for experiencing food craving (Hill, 2007). What seems specific of food craving is the conflict or ambivalence shown toward the desired food in the food craving experience (Rogers and Smit, 2000). As stated by Rogers and Smit (2000, p. 9), “attempting to resist the desire to eat chocolate only causes this desire to become more prominent (salient), and in turn, the experience is labeled as craving, rather than say hunger.”

Food cravings are commonly experienced by most people (Hill, 2007). Some authors suggested that the use of inclusive or exclusive definitions of food craving has a substantial impact on prevalence rates, ranging from 58% (e.g., experiencing any craving) to 4% (e.g., experiencing strong cravings, not in the context of pregnancy, with three or more core features) in a sample of women (Gendall et al., 1997). Significant differences between male and female prevalence rates have also been found in other studies (e.g., Lafay et al., 2001), in which the prevalence rate for food craving was more than twice as high in women (i.e., 28%) than in men (i.e., 13%).

Food cravings have been positively associated to consumption of specific craved foods (Martin et al., 2008; Chao et al., 2014), to body mass index (Burton et al., 2007), to loss of control over eating (Moreno et al., 2009), and to food addiction symptoms (Meule and Kubler, 2012). Some studies revealed that momentary food cravings were moderately correlated with negative affect (e.g., Meule et al., 2012), whereas others found that cognitive reappraisal significantly reduced self-reported desirability of craved foods (Giuliani et al., 2013), thus indicating that both negative affect and reappraisal may be considered as important validation criteria. Another study showed that food cravings scores were significantly higher in people with bulimic eating disorders than in healthy controls (Van den Eynde et al., 2012). Although a number of studies suggested that food craving precipitates binge eating, not all food cravers developed disordered eating (Gendall et al., 1998).

The assessment of food craving mostly relies on self-report instruments. Food craving instruments include both measures of craving for specific foods and general craving scales. An example of the former is the Food Craving Inventory (FCI, White et al., 2002), which measures the frequency of subjective and behavioral cravings for 47 different foods (e.g., fried chicken, chocolate, cereals, French fries, etc.). The craving record (Hill and Heaton-Brown, 1994) is an example of general craving scale based on self-monitoring, which is intended to be completed as soon as possible after the food craving. It consists of 18 questions assessing subjective experiences of food cravings, regardless of whether or not participants eat craved foods.

A significant contribution to the definition and measurement of food craving comes from a multi-faceted approach to the study of the construct (Cepeda-Benito et al., 2000a,b). Two generalized and multifactorial food craving instruments (e.g., the Food Craving Questionnaire-State, FCQ-S, 15 items, and the Food Craving Questionnaire–Trait, FCQ-T, 39 items) have been proposed to assess both short-term changes in food craving (i.e., cravings as a psychological state in response to specific situations) and habitual food cravings (i.e., cravings as a psychological trait; Cepeda-Benito et al., 2000a,b). These instruments investigate multiple aspects of food cravings (e.g., behavioral, cognitive, and physiological ones); they do not refer to craving for a specific food chosen *a priori* by the researcher (e.g., chocolate), but assess cravings for specific foods chosen by the responder. Both the FCQ-S and FCQ-T have shown excellent reliability (αs = 0.97 and 0.94 for FCQ-T and FCQ-S, respectively), and adequate fit for the 9-factor model of the FCQ-T and the 5-factor model of the FCQ-S (Cepeda-Benito et al., 2000b).

The FCQ-T and the FCQ-S have now been translated and validated in different languages, including Spanish (Cepeda-Benito et al., 2000a), Dutch (Nijs et al., 2007), Korean (Noh et al., 2008), and German (Meule et al., 2012). The Spanish study demonstrated the factorial similarity across the Spanish and English versions of the FCQ-T and FCQ-S, in particular the measurement equivalence for the FCQ-T and the partial invariance for the FCQ-S (Cepeda-Benito et al., 2000a). The Dutch study assessed the psychometric properties of the modified versions of the FCQ-T and FCQ-S by developing an index of general food craving (G-FCQ-T and G-FCQ-S, respectively): results indicated that the G-FCQ-T and G-FCQ-S were both reliable and valid measures of trait and state food craving as general dimensions (Nijs et al., 2007). However, the 4-factor solution of the G-FCQ-T that emerged from this research was different from that one proposed in earlier works (Cepeda-Benito et al., 2000a,b). The German study demonstrated that FCQ had good psychometric properties, although with a fewer number of factors (Meule et al., 2012). More importantly, the results of this research showed that FCQ-T-subscales were able to discriminate between individuals who are successful and those who fail in dieting.

Other studies confirmed the multidimensional conceptualization of trait and state food cravings (e.g., Cepeda-Benito et al., 2003). The FCQ-T has also been adapted for the assessment of craving for chocolate (Rodríguez et al., 2007). Moreover, a validation of the FCQ-T and FCQ-S in a clinically heterogeneous sample of eating disorder patients has also been published (Moreno et al., 2008).

More recently, a short version of the FCQ-T (FCQ-T-r) has been developed and validated capturing the core components of food craving, i.e., the emotional and environmental aspects that trigger food craving, the mental images and cognitive processes related to food craving, and the behavioral consequences in terms of searching for and consuming food (Meule et al., 2014). The results of this study suggested that FCQ-T-r has a one-factor structure and high internal consistency. Other works confirmed the good psychometric properties of the FCQ-T-r (e.g., Rodríguez-Martín and Molerio-Pérez, 2014).

The purpose of the present study is to evaluate the reliability and validity of an Italian adaptation of the FCQ-T-r in two independent community samples. As further validation of the use of the FCQ-T-r, we analyze relations between the FCQ-T-r and other important psychological variables (e.g., disordered eating attitudes and behaviors, emotion regulation strategies, positive and negative affective states), which are supposed to be related to food craving on the basis of the literature reviewed above.

## Materials and Methods

### Sample 1

A total of 368 participants aged 18–65 years (*M* = 28.40, SD = 8.77; 79.3% females), living in the center of Italy, voluntarily participated in a study aimed at evaluating the relationships among personality characteristics, emotions, and eating attitudes and behaviors. Trained interviewers recruited potential participants from the general population. Before obtaining verbal consent, participants received information about the aims and characteristics of the study. All participants provided verbal informed consent according to the Helsinki declaration. Those who agreed, completed a set of questionnaires and provided socio-demographic characteristics (e.g., gender, age, education, marital status, and so forth). The subjects were informed that the participation would have taken about 30–40 min and that they had the right to quit the study at any moment.

### Sample 2

Participants were 246 individuals from the general population, aged 20–58 years (*M* = 36.46, SD = 9.92, 49.2% females). Sample 2 characteristics were similar to those of Sample 1 in terms of places of recruitment, questionnaire completion time, and information about the aim of the study. This was a convenience sample that had an established quota of participants by age and gender that were defined according to the Italian population pyramid. Sample 2 has been used in this study as a validation sample. All participants provided verbal consent according to the Helsinki declaration. **Table 1** reports sample descriptive statistics for both samples.

**Table 1:** Sociodemographic characteristics of the samples.

	Sample 1 (*N* = 367)						Sample 2 (*N* = 246)					
	Males		Females		Total		Males		Females		Total	
	*N*	%	*N*	%	*N*	%	*N*	%	*N*	%	*N*	%
**Age category**												
18–29	48	63.2	233	80.1	281	76.6	37	29.6	40	33.1	77	31.3
30–39	18	23.7	46	15.8	64	17.4	44	35.2	40	33.1	84	34.1
40–65	10	13.2	12	4.1	22	6	44	35.2	41	33.9	85	34.6

### Measures

#### Food Craving

Participants’ trait food craving was assessed with 15 items from the original version of the FCQ-T (Cepeda-Benito et al., 2000a; Meule et al., 2014). The FCQ-T was translated into Italian by the last author (senior) of the present paper, and then backtranslated into English by a British native speaker. Comparison of the backtranslation with the original English version revealed no discrepancies. The FCQ-T-r comprises five items from the *Lack of control over eating* (i.e., 2, 3, 25, 26, 29) and the *Thoughts or preoccupation with food* (i.e., 6, 8, 27, 31, 32) subscales, two items from the *Having intentions and plans to consume food* (i.e., 5, 18) and the *Emotions that may be experienced before or during food craving or eating* (i.e., 20, 33) subscales, and one item from the *Cues that may trigger food craving* (i.e., 35) subscale. For each item, participants rated the extent to which each statement would be true for them in general, from 1 (*never*) to 6 (*always*). A sample item is “When I crave something, I know I won’t be able to stop eating once I start.”

#### Disordered Eating Attitudes and Behaviors

Participants rated their levels of disordered eating attitudes and behaviors using the Disordered Eating Questionnaire (DEQ; Lombardo et al., 2004). The DEQ is a 24-item scale consisting of two sections. The first section (disordered eating behaviors) contains 14 items referring to dieting, and concerns about body weight, body shape, and calories. Participants were requested to indicate the frequency with which they experienced each of the symptoms during the last three months. Answers were given on a 6-point scale from 0 (*never*) to 5 (*more than once a day*). A sample item is “(How much you have) reduced the intake of food or calories in order to lose weight.”

The second section (disordered eating attitudes) contains six items referring to the discomfort with body weight and shape, intrusive thoughts (about calories, food, or one’s body), and self-esteem dependent on body weight and shape. Participants were asked to indicate the intensity of these feelings experienced during the last three months on a 7-point scale from 0 (*not at all*) to 6 (*absolutely*). A sample item is “(How much you have) felt your self-esteem was influenced (positively or negatively) by thoughts about your intake of food and calories.” Two validation studies (Lombardo et al., 2004, 2011) evidenced that all the items of both sections loaded onto one factor and, in this way, could be summed in order to compute a total score, notwithstanding the different response scales. Four additional questions on purging behaviors [sample item is “(How much you have) took diuretics to keep your weight under control,” with the same frequency scale as the first section], as well as two questions on self-reported body weight and height, are included in the DEQ.

#### Emotion Regulation Strategies

Participants’ emotion regulation processes were assessed with the Emotion Regulation Questionnaire (ERQ), a questionnaire developed for measuring the extent to which participants use different strategies of emotional control (Gross and John, 2003). The ERQ contains 10 items that form two domain scales: expressive suppression and cognitive reappraisal. Expressive suppression is an emotion regulation strategy that consists in the inhibition of one’s emotion-expressive behavior, whereas cognitive reappraisal is an emotion regulation strategy that targets the meaning or the self-relevance of an emotion eliciting situation (e.g., Gross, 2015). Individuals reported the frequency with which they generally experienced each item on a 7-point scale, from 1 (*strongly disagree*) to 7 (*strongly agree*). Two sample items are “When I want to feel less negative emotion (such as sadness or anger), I change what I’m thinking about” (cognitive reappraisal) and “I control my emotions by not expressing them” (expressive suppression).

#### Affective States

The trait form of the Positive and Negative Affect Schedule (PANAS; Watson et al., 1988) was used to assess participants’ positive and negative affectivity, with 10 affective adjectives measuring positive affect (PA) and 10 affective adjectives measuring negative affect (NA) on 5-point Likert-type scales, from 1 (*never/almost never*) to 5 (*always/almost always*). Examples of these include “active,” “attentive,” and “strong,” from the PA scale, and “scared,” “afraid,” and “guilty,” from the NA scale.

### Data Analyses

Different research approaches were used for this study. First, we applied exploratory factor analysis (EFA) to assess possible underlying dimensions of FCQ-T-r. Subsequent to the EFA, we used confirmatory factor analysis (CFA) and structural equation modeling (SEM) with Mplus 7.2 (Muthén and Muthén, Muthén and Muthén, 1998–2012) to test parameters and goodness of fit of the hypothesized factor model. A robust maximum likelihood method of estimation was applied to test the hypothesized model because, as we will show later, items presented non-negligible violations of normality (Chou and Bentler, 1995).

To evaluate the goodness of fit of the model, several indices were used in the present study: χ^2^, Standardized Root Mean Square Residual (SRMR), Root Mean Square Error of Approximation (RMSEA), and Comparative Fit Index (CFI). The χ^2^ is a test of model misspecification that assesses the difference in magnitude between the sample and the model estimated variance/covariance matrices. A significant χ^2^ indicates a poor fit of the model to the data, whereas a non-significant χ^2^ suggests that a model fits the data well. The χ^2^ statistic has some limitations, such as sensitivity to sample size and to violations of the assumption of multivariate normality. Several fit indices have been proposed to address the limitations of the χ^2^ statistic. One of the most often used indices is the RMSEA. RMSEA is a parsimony-adjusted index that takes into account model complexity (Kline, 2011). It represents the discrepancy of the hypothesized model covariance matrix from the population covariance matrix. Values of RMSEA less than or equal to 0.05 indicate a good fit, values greater than 0.05 but less than or equal to 0.08 indicate an adequate fit, and values greater than 0.10 indicate a poor fit (Browne and Cudek, 1993). A usual practice in SEM literature is to provide the 90% confidence interval (CI) for the RMSEA, which includes the sampling error associated with the estimated RMSEA. A lower bound of 90% CI less than 0.05, as well as an upper limit less than 0.08, indicate a good fit, whereas a maximum upper bound of 0.10 indicates an acceptable fit (Browne and Cudek, 1993). The SRMR is a standardized version of the Root mean square residual and it represents the average residual value derived from the fitting of the model covariance matrix to the sample covariance matrix. SRMR values equal to or less than 0.08 are considered good (Hu and Bentler, 1999). The CFI is an incremental fit index that measures the improvement of overall fit of the model by comparing the hypothesized model with a more restricted one, which specifies no relations among variables. Values of CFI greater than or equal to 0.95 indicate a good fit, values less than 0.95 but greater than 0.90 indicate an adequate fit, and values less than 0.90 indicate a poor fit (Whitley and Kite, 2013).

## Results

### Descriptive Statistics

Food Craving Questionnaire-Trait items presented non-negligible skewness and kurtosis in both samples. In fact, in the first sample skewness ranged from 0.388 to 2.099 with a mean of 1.256 (SD = 0.565), and kurtosis ranged from -0.617 to 3.963 with a mean of 1.532 (SD = 1.633). In the second sample skewness ranged from 0.913 to 2.209 with a mean of 1.487 (SD = 0.442), and kurtosis ranged from 0.008 to 4.986 with a mean of 2.019 (SD = 1.559).

The FCQ-T-r total scores reported in the present study were 32.33 (SD = 12.76) and 28.01 (SD = 12.77) for the first and the second sample, respectively. Furthermore, the total score reflected a non-perfect normality as its kurtosis and skewness are equal to 1.431 and 2.687, and 1.517 and 2.417, respectively, in the first and in the second sample. **Table 2** reports FCQ-T-r scores in the two samples, broken down by gender and age.

**Table 2:** FCQ-T-r scores.

	Sample 1 (*N* = 345)						Sample 2 (*N* = 245)					
	Males		Females		Total		Males		Females		Total	
	Score	SD	Score	SD	Score	SD	Score	SD	Score	SD	Score	SD
**Age category**												
18–29	28.44	9.04	32.89	12.89	32.20	12.46	27.97	12.96	32.23	11.18	30.18	12.18
30–39	29.94	12.50	35.84	14.18	34.10	13.87	24.02	11.92	28.30	14.79	26.06	13.45
40–65	34.38	16.98	24.18	7.21	28.47	12.95	24.66	11.75	31.60	12.25	27.96	12.42
all	29.55	11.13	33	13.05	32.33	12.76	25.42	12.19	30.71	12.84	28.01	12.77

*SD, Standard deviation*.

### Exploratory and Confirmatory Factor Analysis

First, we tested different FCQ-T-r models in both groups with two, three, four, and five latent factors using EFA. Although the fit indices improved as we added underlying latent factors, better fit indices for multidimensional factorial solutions were, in this case, merely a statistical artifact. In fact, the models with multifactorial structure could not be interpreted, because different items of the instrument loaded on more than one factor and, in particular, none of the extracted factors was reasonable from a substantive standpoint. Thus, according to the results of EFA and consistent with previous studies reporting a unidimensional factor structure for the FCQ-T-r (e.g., Meule et al., 2014), we tested a CFA model which assumed a single latent variable and 15 empirical indicators. CFA was conducted using Mplus 7.2. As noted before, due to non-normality of the observed variables (i.e., scale’s items), robust Maximum Likelihood MLMV estimators were used to perform the CFA (Finney and DiStefano, 2006). “MLMV estimators are maximum likelihood parameter estimates with SEs and a mean- and variance-adjusted chi-square test statistic that are robust to non-normality” (Muthén and Muthén, Muthén and Muthén, 1998–2012, p. 603). The hypothesized one-factor model was fitted to both samples data. In sample 1, this model provided a poor fit to the data, showing the following fit indices; χ^2^ (90, *N* = 346) = 300.48, *p* < 0.001; RMSEA = 0.082, 90% CI [0.072; 0.093], *p* < 0.001; CFI = 0.857; SRMR = 0.060. Similarly, a poor fit to the data was provided by the one-factor model also in sample 2 as the fit indices were the following: χ^2^ (90, *N* = 245) = 196.69, *p* < 0.001; RMSEA = 0.070, 90% CI [0.056; 0.083], *p* < 0.001; CFI = 0.853; SRMR = 0.066.

An inspection of wording and content of the Italian translation of the items suggested that there were large similarities among items. Thus, it was reasonable to hypothesize that there may be correlations among these like items even after removing the variance associated with the construct. Furthermore, one could hypothesize a better fitting model that accounts for the wording across items and the common theoretical domain by allowing covariances between the residuals (i.e., the variance in each item not accounted for by the underlying construct; see Ullman, 2006). As suggested by many authors, cross-validation with a different sample is recommended if model modifications are carried out (Ullman, 2006; Weston and Gore, 2006; Tabachnick and Fidell, 2007; Chou and Huh, 2012).

To test a competing theoretical measurement model, based on the hypothesis that other characteristics of the items account for a significant amount of variance in the model, we allowed the residual variances between similarly worded items to covary. Thus, the test of this hypothesis was carried out in both samples by estimating a new model with 14 residual covariances added. We included into the model the error covariance between the following Items: 3 and 2, 32 and 31, 35 and 25, 8 and 6, 27 and 3, 26 and 2, 33 and 20, 29 and 5, 27 and 5, 33 and 27, 32 and 20, 27 and 18, 29 and 18, 26 and 18. As expected, the model, including the correlated residuals, had an acceptable fit in both samples. More specifically, in sample 1 the competing measurement model showed an adequate fit to the data as the fit indices were the following: χ^2^ (76, *N* = 346) = 166.03, *p* < 0.001; RMSEA = 0.059; 90% CI [0.046; 0.071], *p* = 0.119; CFI = 0.939; SRMR = 0.044. Similarly, in sample 2 this model also provided an acceptable fit to the data as the fit indices were the following: χ^2^ (76, *N* = 245) = 114.71, *p* < 0.01; RMSEA = 0.046; 90% CI [0.027; 0.062], *p* = 0.651; CFI = 0.947; SRMR = 0.048. Furthermore, in both samples the χ^2^ statistic, the RMSEA and the SRMR were smaller, and the CFI was larger than the original model. We used a chi-square difference test to verify if the model with the correlated residuals (i.e., the less parsimonious one) was significantly better than the original model (i.e., the model with more constraints). The chi-square difference test (performed using the DIFFTEST facilities in Mplus) was significant in both samples, suggesting that the inclusion of the covariances between each commonly worded item significantly improved the model (sample 1: χ^2^_diff_ (14) = 171.71, *p* < 0.001; sample 2: χ^2^_diff_ (14) = 114.60, *p* < 0.001). **Table 3** shows that the factor scores were all higher than 0.52 (range 0.528–0.852) for sample 1 and.44 (range 0.444–0.864) for sample 2.

**Table 3:** Factor loadings of FCQ-T-r.

Item no.	Sample 1	Sample 2
I2	0.637	0.584
I3	0.679	0.584
I5	0.608	0.766
I6	0.747	0.658
I8	0.614	0.444
I18	0.592	0.743
I20	0.526	0.742
I25	0.601	0.851
I26	0.853	0.864
I27	0.833	0.857
I29	0.835	0.845
I31	0.734	0.856
I32	0.758	0.825
I33	0.598	0.616
I35	0.532	0.669

*Item numbers correspond to the original 39-item version*.

### Reliability and FCQ-T-r Total Score

As coefficient alpha may produce inaccurate estimates of reliability when item errors are correlated, we decided to apply composite reliability which is a test of reliability similar to coefficient omega (Raykov, 1998): in particular, we applied the revised formula for a scale with correlated errors (see Raykov, 2012). Composite reliability was 0.93 for sample 1 and 0.92 for sample 2.

Since sample 2 was considered as the *validation* sample, we used this sample to conduct a series of one-way ANOVA tests on FCQ-T-r. Women had higher FCQ-T-r scores (*M* = 30.71, SD = 12.84) than men (*M* = 25.42, SD = 12.19, *F* = 10.95, *p* < 0.001). No age differences were found in FCQ-T-r. Then, we compared the FCQ-T-r total scores reported in our studies with those resulting from similar studies conducted both in community and clinical samples. The FCQ-T-r scores assessed in our studies were lower than those reported in German, *t*(566) = -9.54, *p* < 0.001, and Spanish community studies, *t*(1484) = -17.16, *p* < 0.001 (Meule et al., 2014; Rodríguez-Martín and Molerio-Pérez, 2014), as well as in the Italian clinical sample, *t*(747) = -12.58, *p* < 0.001 (Innamorati et al., in press).

### Concurrent Validity

The relationship between FCQ-T-r and other psychological variables were assessed by standard Pearson correlation in sample 2 (see **Table 4**). FCQ-T-r was positively and significantly related to DEQ (*r* = 0.575; *p* < 0.001) and negative affect (*r* = 0.275; *p* < 0.001) scores.

**Table 4:** Relationship between the FCQ-T-r and other variables.

	Positive affect	Negative affect	Reappraisal	Expressive suppression	Disordered eating
FCQ-T-r	0.091	0.275^∗∗^	-0.114	-0.060	0.575^∗∗^

*^∗∗^*p* < 0.001*

## Discussion

The FCQ-T is a well-established multidimensional measure of trait food craving. A growing body of literature on FCQ-T psychometric properties has showed its validity and reliability (Cepeda-Benito et al., 2000a; Nijs et al., 2007; Noh et al., 2008; Meule et al., 2012), as well as its utility in clinical contexts (Moreno et al., 2008, 2009). More recently, a short form of the FCQ-T (i.e., the FCQ-T-r) has been proposed (Meule et al., 2014), and two studies conducted with general (Rodríguez-Martín and Molerio-Pérez, 2014) and clinical populations (Innamorati et al., in press) confirmed the validity and reliability of this measure. The goal of the present work was to expand on the above research by providing a CFA study of the FCQ-T-r.

The unidimensional model showed an acceptable fit to the data in both samples. Thus, the current research confirmed the factor structure of the measure in line with previous studies (Meule et al., 2014; Rodríguez-Martín and Molerio-Pérez, 2014; Innamorati et al., in press) and showed that our FCQ-T-r Italian version has an excellent reliability. To our knowledge, this is the first study reporting a cross-validation of the FCQ-T-r using CFA.

Descriptive statistics were carried out to compare FCQ-T-r scores in different age and gender groups. According to previous studies, results showed that women had higher FCQ-T-r scores than men in both samples (Cepeda-Benito et al., 2003; Meule et al., 2014; Rodríguez-Martín and Molerio-Pérez, 2014; Innamorati et al., in press). Furthermore, differently from a community study that showed a positive correlation between age and FCQ-T-r (Rodríguez-Martín and Molerio-Pérez, 2014), no age differences were found for FCQ-T-r scores, in line with previous studies (Meule et al., 2014; Innamorati et al., in press).

Further analyses have been carried out in the second sample in order to evaluate relationships among FCQ-T-r and other relevant measures of disordered eating, emotion regulation strategies, and negative affective states. High positive correlations have been found between trait food craving and disordered eating. This finding is consistent with previous studies which reported concurrent validity coefficients of the FCQ-T-r with low dieting success (Meule et al., 2014), binge eating severity (Innamorati et al., in press), food thought suppression (Rodríguez-Martín and Molerio-Pérez, 2014), and disinhibition and hunger dimensions of the EI (Cepeda-Benito et al., 2000b). Moreover, moderate positive correlations have been found between trait food craving and measures of negative affectivity, according to previous works (e.g., Meule et al., 2012).

Our findings also showed a small and marginally significant negative correlation between trait food craving and cognitive reappraisal. To the best of our knowledge, few studies have showed a possible relationship between food craving and emotion regulation. For example, a study revealed that cognitive reappraisal significantly reduced self-reported desirability of craved foods (Giuliani et al., 2013). Although in the current research this relationship only approached significance, our findings indicated that reappraisal (i.e., a cognitive emotion regulation strategy) could be a possible mediator in the relation between the tendency to crave for food and the subsequent correlated behavior of consuming craved food. Future studies with a longitudinal design should address this issue.

In sum, the present work confirmed the unidimensional model of trait food craving, in line with previous research. In spite of the strengths of this study, there are some limitations to our conclusions. Our sampling method was non-probabilistic, although the second sample was stratified by gender and age according to the national census. Future studies with larger samples and with probabilistic sampling procedures will be useful to avoid bias in estimating FCQ-T-r scores. Only self-report measures were used to assess trait food craving. To avoid that the explained variance could be somewhat inflated by common method effects, future research could use behavioral measures in addition to questionnaires. Moreover, future studies could be extended to other age groups (e.g., children and adolescents), taking into account that they are possible targeted groups for cognitive interventions aimed at preventing food craving and promoting healthy eating attitudes and behaviors (e.g., Silvers et al., 2014).

Despite these limitations, the results of the present study showed that our FCQ-T-r Italian version has good psychometric properties. Thus, it could be used to assess trait food craving in an Italian community population of adultsas a brief and valid alternative to the full version of the FCQ-T.

## Author Contributions

CL designed the study, LI and CB analyzed and interpreted the data, LI wrote the manuscript, CL and CB aided in manuscript revision. All authors read and approved the final manuscript.
